# Paclitaxel-Induced Cutaneous Lupus Erythematosus and Raynaud's Phenomenon

**DOI:** 10.7759/cureus.50974

**Published:** 2023-12-22

**Authors:** Kaveri Rusia, Bhushan Madke, Yash Kashikar, Soham Meghe

**Affiliations:** 1 Dermatology, Jawaharlal Nehru Medical College, Datta Meghe Institute of Higher Education and Research, Wardha, IND

**Keywords:** drug-induced lupus, raynaud's phenomenon, secondary raynaud's phenomenon, chemotherapy-induced, chemotherapy toxicities

## Abstract

Taxanes, in combination with platinum-based drugs, are considered the initial treatment option for certain types of cancer, including ovarian cancer. Here, we report the case of a 59-year-old woman who developed a malar rash on her face, a maculopapular rash on her forearms, and bluish discoloration on her fingers immediately following the end of the third cycle of chemotherapy. After discontinuing paclitaxel and using oral and topical steroids for rash and diltiazem and topical minoxidil for the treatment of Raynaud's phenomenon, the symptoms completely resolved. While taxanes are known to cause drug-induced lupus, there has never been any information on taxanes causing isolated Raynaud's phenomenon. This is the first case report that suggests paclitaxel-induced Raynaud's phenomenon along with paclitaxel-induced lupus.

## Introduction

Anti-cancer medications used for ovarian cancer that are frequently recommended include taxane substances like paclitaxel and docetaxel. They stabilize the microtubule by enhancing the polymerization of tubulin and inhibiting their depolymerization. There have been reports of a multiple variety of skin adverse effects caused by taxanes, such as scleroderma-like skin lesions, photosensitive cutaneous lupus erythematosus, nail changes, mucosal involvement, dysgeusia, alopecia, and hand-foot syndrome [1].

Several lupus-like syndromes are caused by various types of drugs and resolve when the drug is discontinued; this is known as drug-induced lupus erythematosus (DILE). The skin lesions of subacute cutaneous lupus erythematosus (SCLE) have a unique morphology and are linked with autoantibodies, but systemic features are absent. The skin lesions of SCLE are usually annular-polycyclic or papulosquamous and affect symmetrical photo-distributed areas [2].

Raynaud's phenomenon (RP) is a condition that causes temporary constriction of the blood vessels in the fingers and toes. It is often triggered by cold temperatures or emotional stress. Most people (around 80-90%) with RP have primary (also called idiopathic) RP, which means that there is no underlying medical condition that causes it. However, secondary RP can occur as a result of various medical conditions or drug-related causes [3]. Patients receiving bleomycin, alone or with other chemo drugs, are at high risk for RP. It seldom results in considerable functional impairment and usually resolves on its own, especially after the inciting antineoplastic medication is stopped. Nevertheless, it frequently returns after the reintroduction of the agent causing it [4].

## Case presentation

**Figure 1:**
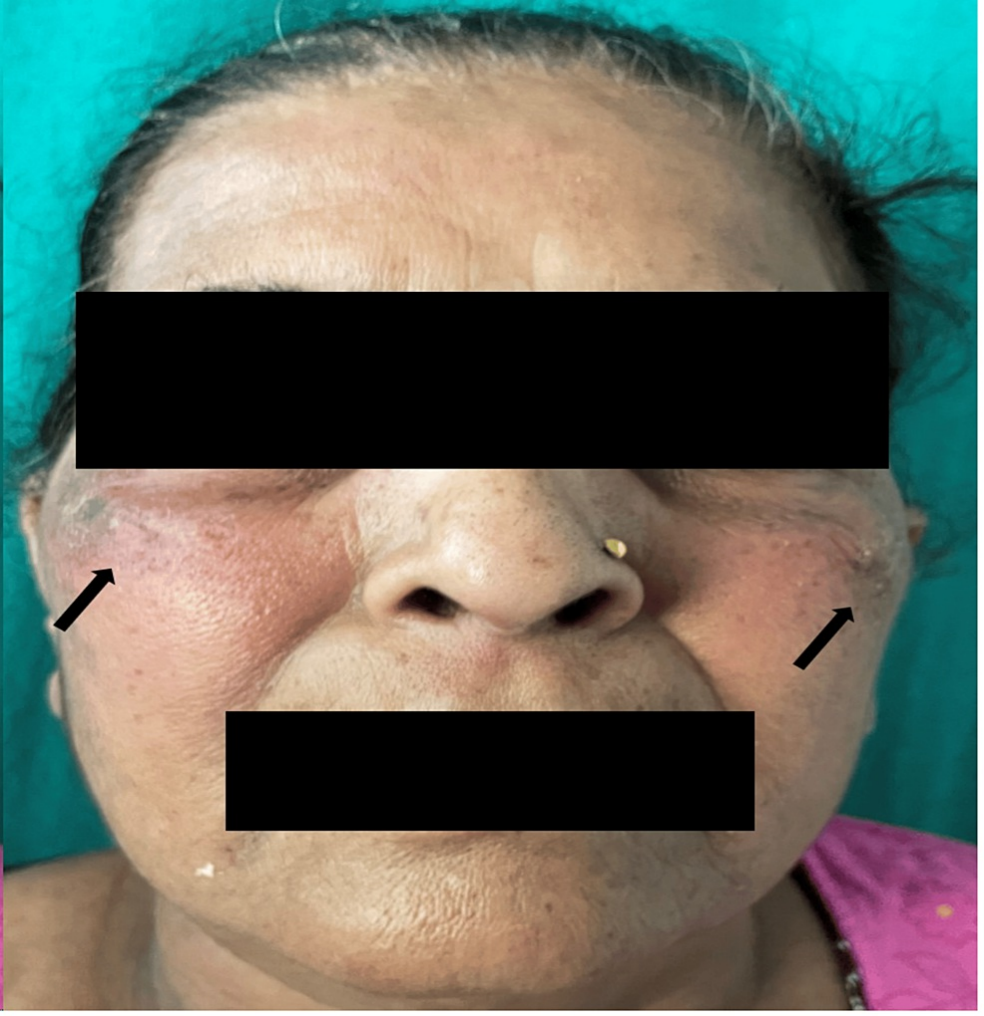
Malar rash on the face (black arrows)

**Figure 2:**
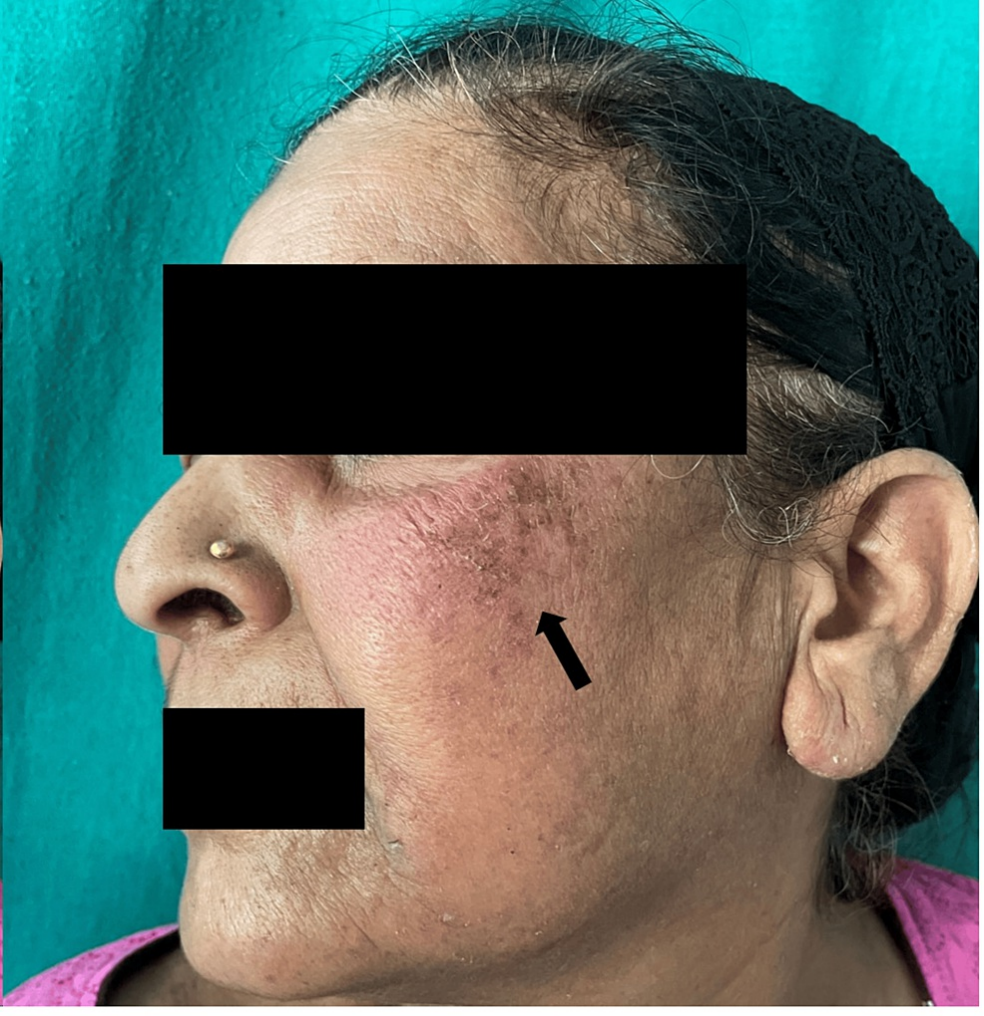
Erythematous patch on the left cheek (black arrow)

**Figure 3:**
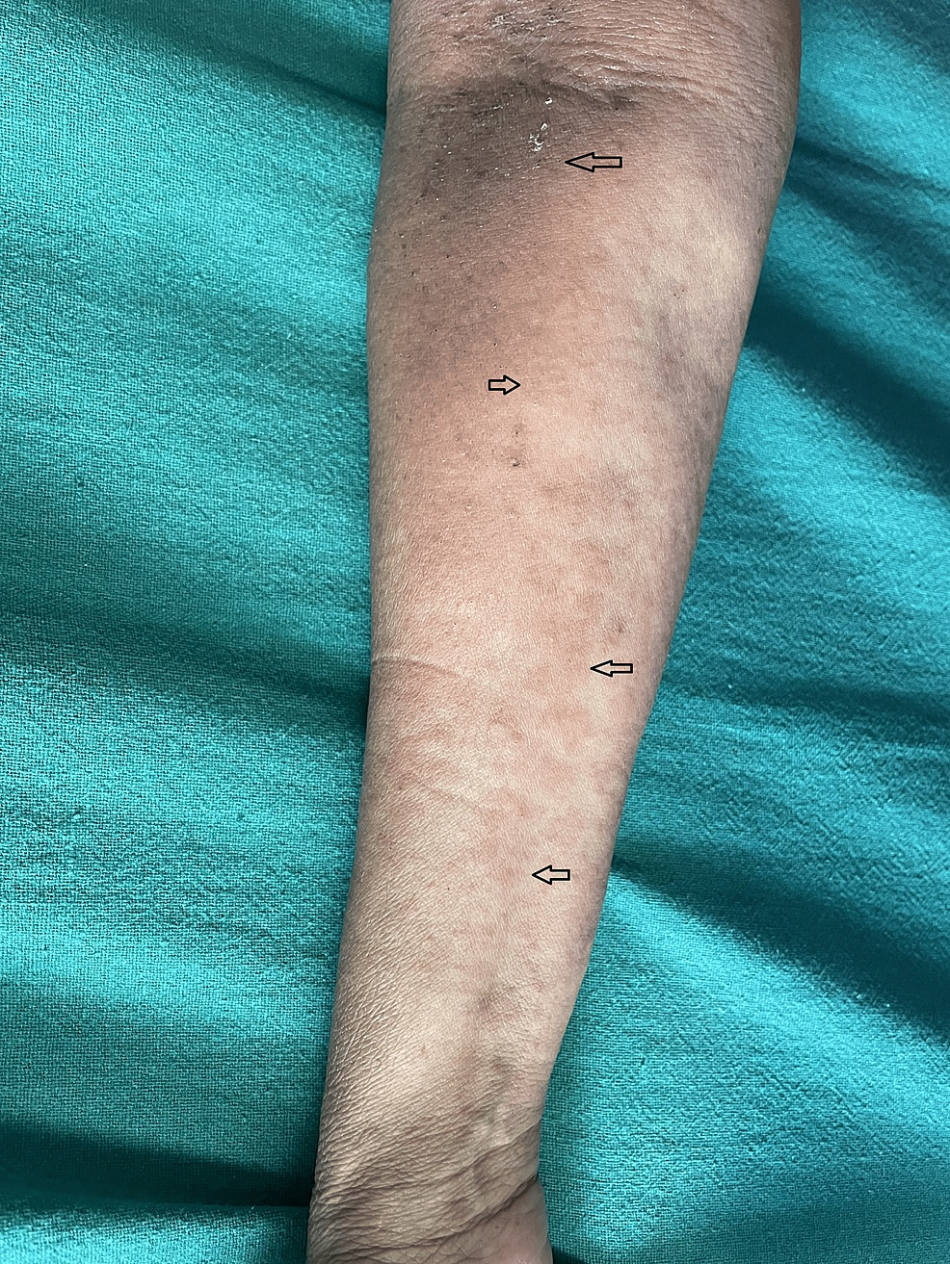
Erythematous macules and papules with scaling present on the left forearm (black arrows)

**Figure 4:**
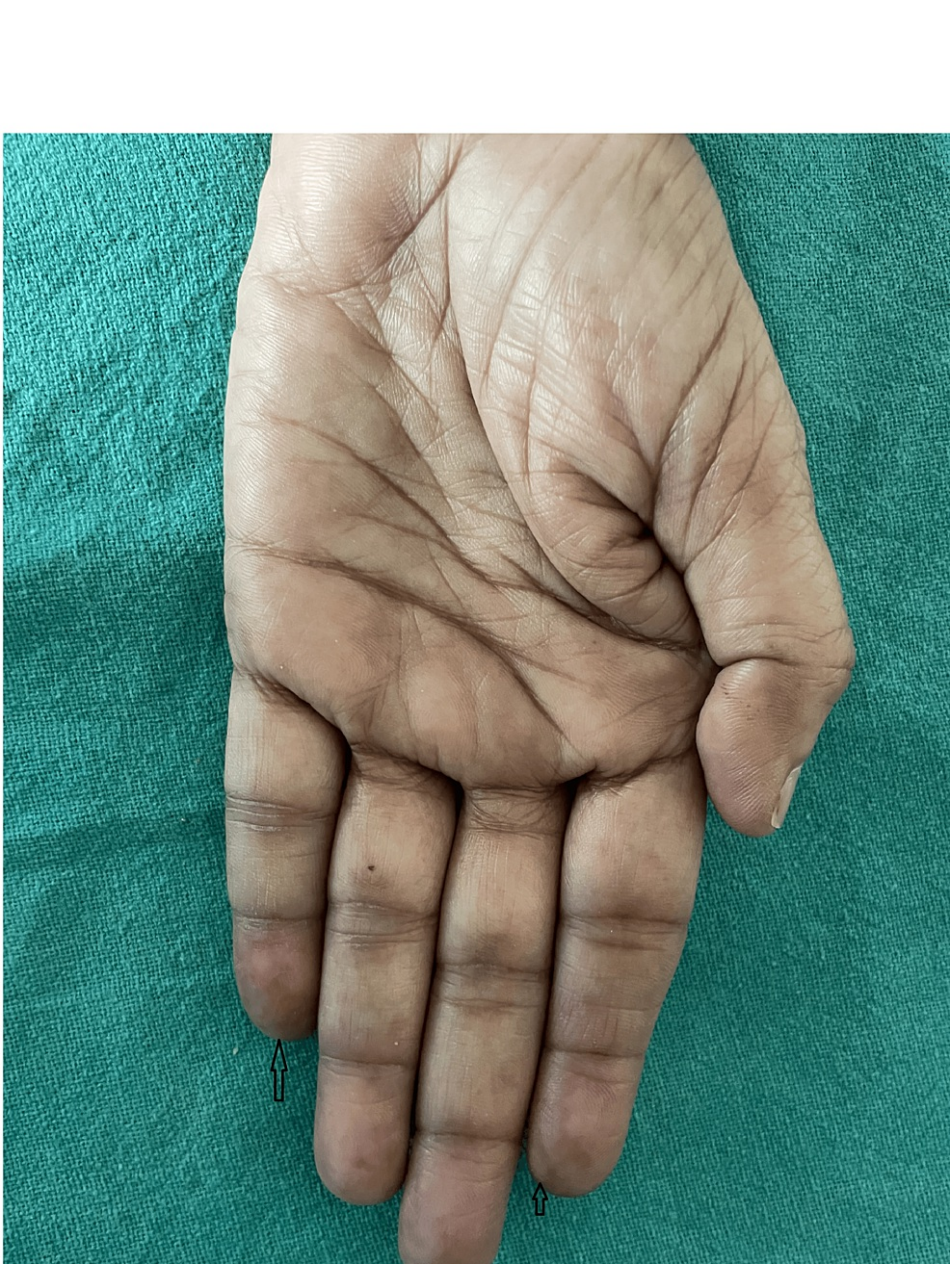
Bluish discoloration of the little and index fingers of the left hand (black arrows)

A 59-year-old woman visited the dermatology outpatient department due to a red rash on her face (Figure 1 and Figure 2) and forearms (Figure 3) and a bluish discoloration on her left little and index fingers (Figure 4).

The rash and discoloration had been present for six days. Upon further inquiry, the patient reported a history of photosensitivity, joint pain, and low-grade intermittent fever. Three months ago, the patient was diagnosed with right ovarian adenocarcinoma with positive BerEP4 and PAX8 immunohistochemistry markers and had received three cycles of chemotherapy, including paclitaxel (120 mg) and carboplatin (200 mg) every two weeks. Within a week of receiving the third cycle of chemotherapy, she noticed the rash on her face, along with photosensitivity. There were no similar complaints in the past. The patient did not report any other symptoms like breathlessness, difficulty swallowing, tightening of skin, digital ulcers, chest discomfort, neurological symptoms, oral ulcers, hematuria, or edema of hands and feet.

During her complete blood count examination, complement levels were within normal range with a raised erythrocyte sedimentation rate (ESR) of 80 mm/min. She had high titers of antinuclear antibodies (ANA) (1:240), but anti-histone antibodies and anti-double-stranded DNA antibodies were negative. Her urine test, liver enzymes, and kidney function test were normal. Nailfold capillaroscopy did not suggest any changes of systemic sclerosis.

**Figure 5:**
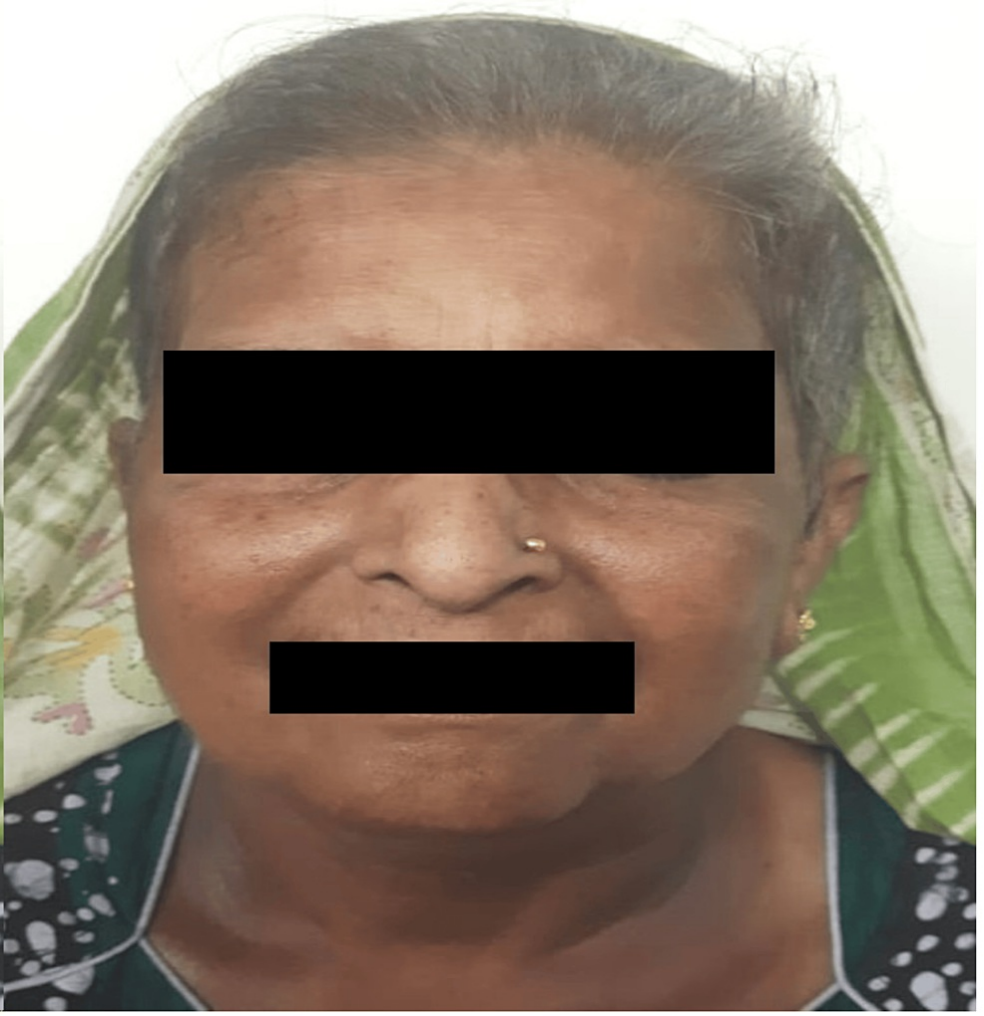
Resolution of malar rash with mild hyperpigmentation of the face

**Figure 6:**
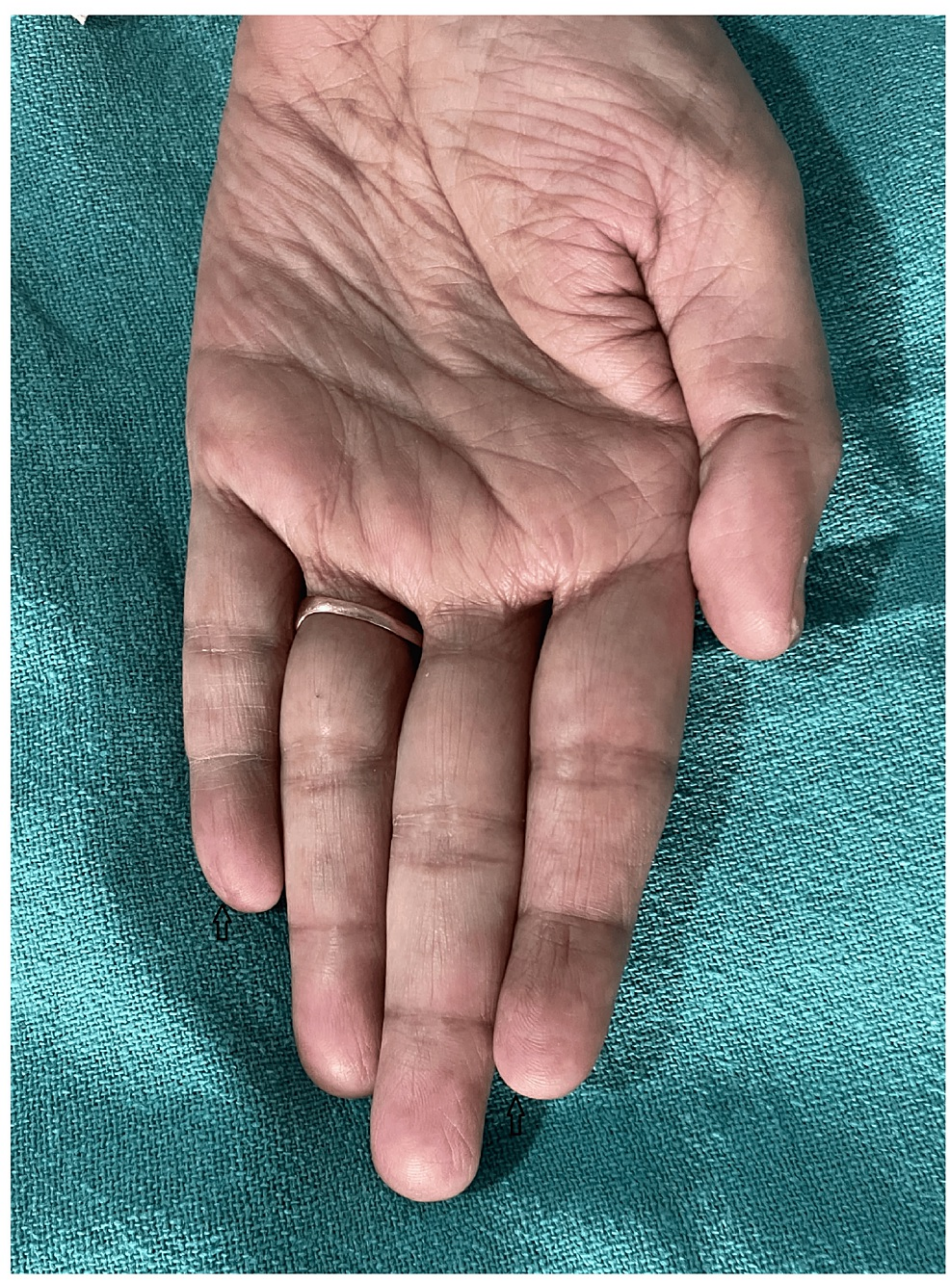
Resolution of RP of the little and index fingers (black arrows) RP: Raynaud's phenomenon

The patient was diagnosed with paclitaxel-induced cutaneous lupus erythematosus and RP based on her history and temporal history of paclitaxel chemotherapy. She was treated with oral prednisolone (20 mg) and topical application of mometasone furoate 0.1% cream for her malar and forearm rash. For RP, the patient was given a tablet diltiazem (30 mg) daily and a topical application of minoxidil 5% gel. There was complete resolution of the rash (Figure 5) and RP (Figure 6) after two months of treatment, leaving behind mild hyperpigmentation.

## Discussion

Paclitaxel belongs to the taxane group of chemotherapy drugs used to treat various types of cancers, including carcinoma of the breast, carcinoma of the ovary, gastrointestinal cancer, and carcinoma of the neck and head. However, treatment with paclitaxel may cause various side effects such as neurological pain, alopecia, and changes in nails although cutaneous disorders like photosensitivity are rare [5].

One of the common manifestations of systemic lupus erythematosus is cutaneous lupus lesions, which typically occur on areas of the skin that are exposed to sunlight. These lesions can be triggered by exposure to sunlight or other sources of UV radiation [5]. Clinically, immunologically, and histopathologically, drug-induced SCLE is identical to idiopathic SCLE. As a result, if there are diffuse cutaneous lesions after the injection of an offending chemical, their remission upon stopping the treatment is indicative of drug-induced SCLE diagnosis [2].

For the diagnosis of cutaneous lupus, it is a must to exclude the history of systemic lupus erythematosus before starting the culprit drug; there should be one or more clinical presentations of systemic lupus erythematosus, resolution of the symptoms after stopping of drug, and positive ANA [6]. In this case, there are the presence of a typical malar rash on the face, photosensitivity, joint pain, and fever along with positive ANA; also, the lesions resolved completely after stopping paclitaxel.

Drug hypersensitivity, serum sickness-like illness, flaring up of already-existing lupus and remission of lupus due to the drug, eosinophilia-myalgia syndrome, hemolytic anemia caused by drugs, toxic oil syndrome, and lupus due to other environmental factors or heavy metals are among various conditions that can cause drug-induced lupus [7]. The pathogenesis of lupus due to drugs is incompletely understood, and it involves environmental factors, sun exposure, and the activation of adaptive as well as humoral immunity which leads to the production of cytokine, thus causing inflammation. The treatment involves stopping the offending drug(s), topical steroids, and calcineurin inhibitors. In a few cases, oral steroids and immunosuppressive drugs are required to treat the moderate to severe adverse effects [8]. We have treated the patient with oral and topical steroids.

Up to 10% of the general population suffer from RP, which is a common condition characterized by episodes or sudden spasms of blood vessels in either the hands, feet, or both. This clinical presentation is sometimes triggered or exacerbated by cold exposure, vibrations, or emotional stress. During an episode, the affected area may first turn pale, then blue, and later red, accompanied by pain [9]. According to a study conducted by Ketpueak et al., 21% of the patients exhibited RP along with edema of the hands and feet. However, in our case, the patient only has RP and no other cutaneous signs of scleroderma [1].

The exact cause of RP caused by cancer treatments is not well understood and likely involves multiple factors. Patients undergoing treatment for testicular cancer often experience RP [10]. One of the reasons is damage to the blood vessels caused by chemotherapeutic drugs, leading to endothelium dysfunction that remains even after treatment. Patients with RP syndrome after chemotherapy have been found to have an enhanced central sympathetic vasoconstrictor reflex and altered non-neurogenic vasomuscular autoregulation [11].

The management of RP begins with addressing the underlying cause, followed by making lifestyle modifications and then administering drugs. The various types of drugs used for treating RP include calcium channel blockers, phosphodiesterase-5 inhibitors, and vasodilators. In our case, we treated the patient with a calcium channel blocker and a topical vasodilator [12].

This is a case of drug-induced lupus and isolated RP caused by paclitaxel. Although uncommon, cutaneous lupus can be a side effect of paclitaxel. While there have been reports of RP caused by multiple chemotherapeutic and non-chemotherapeutic drugs, there has been no single case report of RP caused by paclitaxel.

## Conclusions

Dermatologic adverse effects can be caused by a variety of anticancer medications. This case highlights the rare but potential side effects of paclitaxel, like drug-induced cutaneous lupus erythematosus and RP. It is important to recognize these adverse reactions early, as it can make life better for the patient. Therefore, early identification of these adverse effects is crucial to prevent complications associated with them.
